# Effects of Drug Policy Changes on Evolution of Molecular Markers of* Plasmodium falciparum* Resistance to Chloroquine, Amodiaquine, and Sulphadoxine-Pyrimethamine in the South West Region of Cameroon

**DOI:** 10.1155/2018/7071383

**Published:** 2018-05-02

**Authors:** Marcel N. Moyeh, Dieudonne L. Njimoh, Marie Solange Evehe, Innocent M. Ali, Akindeh M. Nji, Dominique N. Nkafu, Palmer N. Masumbe, Atogho-Tiedeu Barbara, Valentine N. Ndikum, Wilfred F. Mbacham

**Affiliations:** ^1^Department of Biochemistry & Molecular Biology, University of Buea, PB 63, Buea, Cameroon; ^2^Laboratory for Public Health Research Biotechnologies, University of Yaoundé I, BP 8094, Yaoundé, Cameroon; ^3^Department of Biochemistry, University of Yaoundé I, PB 812, Yaoundé, Cameroon; ^4^Department of Biochemistry, University of Dschang, BP 67, Dschang, Cameroon

## Abstract

**Background:**

As a result of the spread of parasites resistant to antimalarial drugs, Malaria treatment guidelines in Cameroon evolved from nonartemisinin monotherapy to artemisinin-based combination therapy. The aim of this study was to assess the effect of these therapy changes on the prevalence of molecular markers of resistance from 2003 to 2013 in Mutengene, Cameroon.

**Methodology:**

Dry blood samples (collected in 2003–2005 and 2009–2013) were used for parasite DNA extraction. Drug resistance genes were amplified by PCR and hybridized with oligonucleotide probes or subjected to restriction digestion. The prevalence of individual marker polymorphisms and haplotypes was compared in these two study periods using the Chi square test.

**Results:**

Alleles conferring resistance to 4-aminoquinolines in the* Pfcrt* 76T and* Pfmdr1* 86Y, 184F, and 1246Y genotypes showed a significant reduction of 97.0% to 66.9%, 83.6% to 45.2%, 97.3% to 56.0%, and 3.1% to 0.0%, respectively (*P* < 0.05). No difference was observed in SNPs associated with antifolate drugs resistance 51I, 59R, 108N, or 540E (*P* > 0.05). Haplotype analysis in the* Pfmdr1* gene showed a reduction in the YFD from 75.90% to 42.2%, *P* < 0.0001, and an increase in the NYD (2.9% to 30.1%;  *P* < 0.0001).

**Conclusions:**

The results indicated a gradual return of the 4-aminoquinoline sensitive genotype while the antifolate resistant genotypes increased to saturation.

## 1. Background

Despite reports of declining global malaria incidence, the WHO African region still registered 90% of the global 216 million malaria cases recorded with about 91% of global 445000 deaths recorded in 2016 [1]. The most vulnerable group of individuals to this dreaded menace still remain children below 5 years, pregnant women, and naïve travellers from nonmalarious areas to malaria endemic areas. This persistence in the incidence may be accounted for by poverty, illiteracy/ignorance, and inadequate medical infrastructure. Malaria and poverty have previously been shown to be intimately linked as most malarious communities are highly impoverished communities [2, 3].

According to reports from the Cameroon Academy of Science, 40% of morbidity, 36% of outpatient consultation, 48% of hospitalization, and 67% of deaths among children below 5 years in Cameroon were caused by malaria [4]. The control measures put in place by the Cameroon national malaria control program to wade off this infection include vector control through deployment of long lasting insecticide treated bed nets (LLIN) and indoor residual spraying (IRS) of homes, the use of intermittent preventive therapy in infants and pregnant women (IPTi and IPTp), accurate diagnosis of diseased cases prior to treatment, and the use of artemisinin-based combination therapy (ACT) for the treatment of infected cases [5].

In Cameroon, malaria treatment guidelines have evolved since 2002 from the use of chloroquine through the use of amodiaquine and sulphadoxine-pyrimethamine to artemisinin-based combination therapy [5]. These swift changes were brought about by the rapid development and spread of resistant parasite strains first to chloroquine [6–8] and later to sulphadoxine-pyrimethamine [9]. Although the mechanism of chloroquine resistance is yet to be completely understood, it has been shown to be positively correlated with mutations in the chloroquine resistance transporter gene* (Pfcrt)* that encodes a vacuolar trans-membrane protein [10, 11]. Mutation in codon 76 (lysine to threonine) that leads to a charge loss in the ensuing protein has been found in all chloroquine-resistant parasite lines and clinical isolates. As such, it is being used as a molecular marker to monitor chloroquine resistance in field isolates [12]. Mutations within the* Plasmodium falciparum* multidrug-resistant* (Pfmdr)1* gene principally the N86Y, Y184F, S1034C, N1042D, and D1246Y were shown to play a modulatory role in further decreasing sensitivity to chloroquine as well as other aminoquinolines such as amodiaquine (AQ) and mefloquine [13, 14]. Resistance to the antifolate drug pyrimethamine results from the accumulation of single nucleotide polymorphisms (SNPs) in the dihydrofolate reductase* (dhfr)* domain of the dihydrofolate reductase-thymidylate synthase* (dhfr*-*ts)* gene [15]. SNPs at positions 108 (S108N), 51 (N51I) and 59 (C59R) [16, 17] are all associated with reduced sensitivity to pyrimethamine. Resistance to sulphadoxine and sulfa drugs arise from the accumulation of mutations at five codons in the dihydropteroate synthase* (dhps)* gene leading to amino acid change S436A, A437G, K540E, A581G, and S436F [18].

The recent gains in global malaria control are threatened by the emergence of artemisinin resistance in South East Asia [19] and the probable spread to sensitive areas. Studies carried out in South Eastern Cameroon [20] as well as in Kenya and Malawi suggested that removal of chloroquine pressure from the population led to gradual reduction in the proportion of circulating mutant genotypes of* Pfcrt* gene and a return to the chloroquine sensitive genotype [21–23]. It was thus predicted that removal of CQ and SP pressure from the Cameroon population and introduction of artesunate-amodiaquine (ASAQ) and artemether-lumefantrine (AL) [5] will lead to a gradual reduction of the CQ and SP resistant genotypes. Therefore, the Government of Cameroon opted for the use of ASAQ and subsequently AL for the treatment of uncomplicated malaria in Cameroon. Return to the sensitive genotype can be assessed by determining the change in the prevalence of drug resistance markers with changing therapy. This study was designed to determine the changes in the prevalence of molecular markers of resistance to 4-aminoquinoline and antifolate drugs as the treatment policy changed from nonartemisinin-based monotherapy to the current artemisinin-based combination therapy in Mutengene, South West Region of Cameroon.

## 2. Methodology

This study was carried out with samples collected from the Baptist Hospital in Mutengene. Mutengene is found along the Atlantic coastline, highly endemic for* P. falciparum* and transmission observed all year round [24]. It falls within the littoral forest geoecological zone located at coordinates 4°5′28′′N and 9°18′51′′E as mapped by Kleinschmidt et al. [25]. The climate is equatorial with an annual rainfall of 10000 mm and average temperature of 25°C. The vegetation is semimangrove and tropical wet forest. Mutengene is bordered to the South and Southeast by the sea and to the North and Northeast by Mount Cameroon.

The study population included male and female children aged between 6 months and 10 years, which resided within 10 km from the Baptist Hospital in Mutengene. Samples used for this study were pretreatment* P. falciparum* infected blood samples collected during two separate clinical trials carried out between 2003 and 2005 and 2009 and 2013. The blood samples confirmed by microscopy as positive with monoinfection for* P. falciparum* were spotted on filter paper, allowed to air dry, and transported to the Laboratory of Public Health Biotechnologies of the University of Yaoundé I for genetic analysis. The results of the therapeutic efficacy and safety of the clinical trials have been reported elsewhere [26, 27].

Genomic DNA was extracted from filter paper (Whatmann® No. 3, Sigma-Aldrich, Germany) stained blood samples by boiling in chelex®-100 (Bio-Rad, Berkeley California, USA) as already described [28]. Nested Polymerase chain reaction amplification of drug resistance gene fragments spanning regions of interest was carried out using sequence specific primers obtained from Inqaba Biotec (Pretoria, South Africa). A fragment of the* Pfcrt* gene spanning codon 76 and* Pfmdr1* spanning codons 86, 184, and 1246 were amplified as described by Djimdé et al. [12]. PCR amplification of the* Pfdhfr* and* Pfdhps* gene fragments spanning codons 51-108 and codons 437-540, respectively, was carried out as described by Plowe et al. [28]. The amplified gene fragments for samples obtained between 2003 and 2005 were analysed by hybridization with labeled sequence specific oligonucleotide probes as previously described [29]. Amplified gene fragments of* Pfcrt and Pfmdr1* in samples obtained between 2009 and 2013 were analysed by restriction fragment length polymorphism as already described [12] while the amplified fragments of the* Pfdhfr* and* Pfdhps* genes were restriction digested as described by Duraisingh et al. [30]. Haplotypes were constructed for each sample by combining the various point mutations for each gene fragment in each sample. These haplotypes were considered resistant haplotypes if they all represented combination of resistant related mutations, sensitive if they represented combinations of wild-type alleles or mixed if both forms were present.

Generated data was entered in excel and exported to GraphPad Prism version 6 (GraphPad Prism, La Jolla, CA) for analysis. Descriptive statistics (means and proportions) were used to analyse baseline characteristics of study participants while differences between group proportions were analysed using chi-square test. Statistical significance was considered at *P* < 0.05. SNPs for each gene fragment were analysed by “custom sorting” alphabetically in excel to produce the various haplotypes for the gene. In the analysis to compare SNPs between the two study periods, mixed infections with both the wild type and the mutant were all considered mutants while, in the construction of haplotypes between the two periods, mixed infections with both the wild type and the mutant were removed from the analysis.

## 3. Ethical Consideration

Ethical approval for the 2003–2005 study was obtained from three major bodies: the Institutional Review Board of the Cameroon Baptist Convention Health Board, the National Ethics Committee of the Ministry of Public health Cameroon, and the Ethics Review Committee of the London School of Hygiene and Tropical medicine. Ethical approval authorizing the conduct of the 2009–2013 study was obtained from the Institutional Review Board of the Cameroon Baptist Convention Health Board, the Institutional Review Board of the Biotechnology Centre of the University of Yaoundé 1, and the World Health Organisation (WHO ERC) Ethical Review Committee.

The informed consent form was clearly written in English, stating all the procedures to be carried out, the duration of participation, and the objectives of the study. The information in the consent form was explained to the participants in pidgin for guardians who could not read or write and a third party (interpreter) was invited for a parent who did not understand pidgin or English. Participants included in the study had to indicate their willingness to participate either by signing the consent form or through a thumb print. Guardians/parents were also informed of their right to withdraw from the study at any time and still benefit from the full course of treatment for their child.

## 4. Results

A total of 238 and 260 samples were analysed 2003–2005 and 2009–2013, respectively. The age range of participants 2003–2005 was 6 months to 5 years with a mean age of 27.6 months and mean weight of 12.4 kg. Of the 238 enrolled participants, 117 were females and 123 males. For participants enrolled in the 2009–2013 study, the age ranged from 6 months to 10 years with a mean age of 57.3 months and a mean weight of 18.6 kg. Of the 260 enrolled participants, 119 were males and 141 were females.

**Figure 1:**
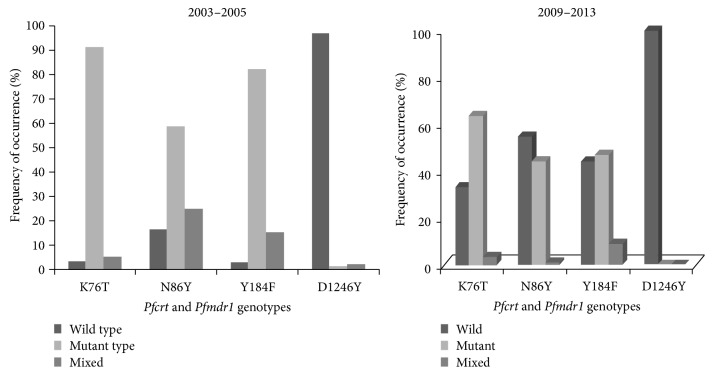
Genotypes of* Pfcrt* and* Pfmdr1* genes in the two study periods.

*P. falciparum* DNA was successfully extracted from all the collected samples. For the samples collected 2003–2005, the* Pfcrt* 76T mutation was observed in 91.7% (211/230) while the mutant 86Y, 184F, and 1246Y of the* Pfmdr1* gene were observed in 58.8% (133/226), 82.1% (183/223), and 1.3% (3/226), respectively. The wild-type K76 allele was observed in 3.0% (7/230) while the N86, Y184, and D1246 alleles were found in 16.4% (37/226), 2.7% (6/223), and 96.9% (219/226), respectively. Mixed infections containing both the wild type and mutant were observed in 5.3% (12/230) of* Pfcrt* and 24.8% (56/226), 15.2% (34/223), and 1.8% (4/226) at codons 86, 184, and 1246 of the* Pfmdr-1* gene, respectively. For the samples collected 2009–2013, the presence of the* Pfcrt* 76T was observed in 63.4% (165/260) and the* Pfmdr1* 86Y mutation was observed in 44.2% (92/208). The* Pfmdr1* 184F genotype reduced to 47.0% (110/234) and the* Pfmdr1* 1246Y mutation was not observed. The wild-type genotype of the* Pfcrt* gene was observed in 33.1% (86/260) whereas the wild-type N86, Y184, and D1246 were found in 54.8% (114/208), 44.0% (103/234), and 100% (250/250), respectively, mixed infections occurred in 3.5% (9/260), 1.0% (2/208), 9.0% (21/234), and 0.0% (0/250) of codons on* Pfcrt* 76 and* Pfmdr-1* 86, 184, and 1246, respectively.  Figure 1 shows the frequency of the* Pfcrt* and* Pfmdr1* genotypes in the two study periods.

Analysis of alleles conferring resistance to antifolate drugs (*Pfdhfr* and* Pfdhps*) showed that the prevalence of mutant alleles was generally high in field samples collected in 2003–2005 (Table 1). This was not the case with 540E mutation that was observed in only 5 samples (2.2%). Between 2009–2013, the prevalence of these antifolate markers increased to near saturation in the population except for the 540E that was not observed. Instead, the wild-type K540 increased to saturation (100%) in the parasite population.

**Figure 2:**
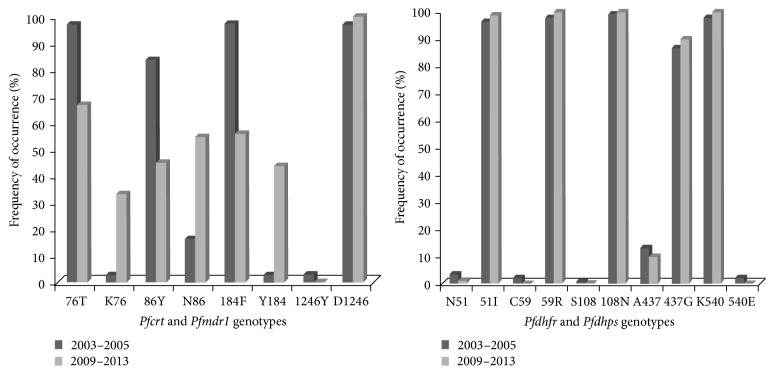
Distribution of wild-type and mutant genotypes between 2003 and 2013.

SNP analysis between the two study periods showed that the proportion of parasites with the mutant allele of* Pfcrt* (T76 genotype) was very high in 2003–2005 (96.1%) but dropped significantly to 67.0% in 2013 (*P* < 0.0001). Similarly, analysis of field isolates showed that the prevalence of the mutant alleles for* Pfmdr1* having the 86Y and 184F genotypes was very high in 2003 (83.6% and 97.3%) but dropped significantly to 45.2% and 56.0%, respectively (*P* < 0.0001) between 2009 and 2013. The antifolate mutant genotypes already near saturation did not change much.  Figure 2 shows the frequency of the mutant and the wild-type genotype between 2003 and 2013 for the 4-aminoquinoline and antifolate genotypes.

The most prevalent haplotype of the* Pfmdr1* gene in the 2003–2005 samples was the YFD [75.9% (129/170)] followed by the NFD [17.1% (29/170)]. The other haplotypes such as the NYD, YFY, and YYY were found in a few isolated cases. Between 2009 and 2013, the YFD haplotype of the* Pfmdr1* gene was still the most common (87/206) but have reduced in prevalence to 42.2% (compared to 75.9% in 2003; *P* < 0.0001). The NYD haplotype increased in prevalence to 30.1% (62/206) compared to 2.7% that was observed in 2003–2005 (*P* < 0.0001). The prevalence of NFD haplotype increased from 17.1% in 2003–2005 to 25.2% (52/206) in 2009–2013 (*P* = 0.08).

Haplotype analysis of the* Pfdhfr* genotypes showed that the triple mutant (CIRN) haplotype was the most predominant in 2003–2005 with the prevalence of 95.9% (213/222). The haplotype of CICN, CNCS, and CNRN was rare. For the* Pfdhps* haplotypes, the wild-type AK was found in 11.6% (26/225) while the GK haplotype was 85.8% (193/225). Only a single sample was observed as the double mutant GE haplotype. Combining the haplotypes of* Pfdhfr* and* Pfdhps*, the CIRN-GK and the CIRN-AK were the most predominant type with 85.6% and 11.9% found in field samples. The quintuple mutant having the triple CIRN and the double GE mutant haplotype was only found in one sample. In field isolates obtained between 2009 and 2013, the prevalence of the CIRN haplotype conferring resistance to pyrimethamine was found in 98.8% (240/243) while the CNRN was 1.2% (3/243). The other haplotypes such as the CICN, CNCN, and the wild-type CNCS were not observed. For the haplotypes of the* Pfdhps* gene involved in sulphadoxine metabolism, only the GK and AK were found in 90.0% (216/240) and 10.0% (24/240).

## 5. Discussion

The results clearly demonstrate a gradual return to the 4-aminoquinoline sensitive genotype of circulating* P. falciparum* populations within the study area. It showed a significant return to the wild-type allele of* Pfcrt* and* Pfmdr1*. The ban on chloroquine importation and use in Cameroon by 2002 and the consequent removal of chloroquine drug pressure from the population could be responsible for the return to the sensitive genotype of these markers. The return to the K76 allele is similar to results obtained by Ndam et al. [20] in South Eastern Cameroon as well as in East African countries such as Kenya [21] and Malawi [22, 23]. The results equally showed that although AQ, a 4-aminoquinoline, was still part of the new ACTs adopted for the treatment of uncomplicated* falciparum* malaria in Cameroon, the prevalence of the SNPs associated with 4-aminoquinoline resistance 76T, 86Y, 184Y, and 1246Y was observed to decrease from 2003 to 2013. These results were consistent with results obtained in Tanzania [31] and Uganda [32]. An increase in* pfmdr1 N86* threatens AL but favours ASAQ which is currently the main first-line drug in use for management of uncomplicated malaria cases in Cameroon. There could be a benefit of rotating first-line medications that have opposite effects on the incidence of markers of resistance, with the ultimate aim of reducing the evolution of these markers. This reduction in the prevalence of circulating mutant genotype and an increase in the prevalence of the wild-type genotype could be a result of high efficacy of ACTs to 4-aminoquinoline resistant parasites and selection of wild-type alleles following treatment with AL [33–35] given that artemether-lumefantrine was fully implemented for the treatment of uncomplicated malaria in Cameroon in 2006 [36]. Amidst fears that the ACT will fail if the level of resistance to the partner drug increases, reduction in the prevalence of amodiaquine resistance markers may be an indication that the therapeutic lifespan of the ACTs may be sustained. Studies by Kublin et al. [22] showed that it took a similar time period for the prevalence of the chloroquine-resistant* Pfcrt* genotype in Malawi to reduce from 85% to 13%. The refractoriness of the decrease in Cameroon might have been due to the inclusion of AQ as part of the ASAQ combination, thus still maintaining some selection pressure for the resistant* Pfcrt* genotype. This drug (ASAQ) is used as the main first-line medication for the treatment of uncomplicated malaria in Cameroon.

The prevalence of the SNPs on the* Pfdhfr* (especially 51I, 59R, and 108N) and* Pfdhps* (437G) was observed to remain high between the study periods. This was consistent with results obtained in Kenya [37] where the prevalence of antifolate markers remained high despite withdrawal of SP from national treatment guidelines. The SP drug pressure in the Cameroon public has been maintained despite the treatment review to adopt ACTs for treatment of uncomplicated malaria. Its single-dose therapy compliance is greatly improved and most patients prefer to revert back to SP instead of the three-day course of ACTs. Also, the Ministry of Health in Cameroon adopted SP for chemoprophylaxis in pregnant women and children below 5 years coupled with the prophylactic treatment of opportunistic infections in HIV infected patients using antifolates such as trimethoprim-sulfamethoxazole. SP was incorporated into the malaria control protocol to serve an intermittent preventive therapy in pregnant women (IPTp) and infants (IPTi). The continued administration of SP in this vulnerable group of patients sustained the drug pressure in the population and thus selection of mutant alleles. The WHO strongly recommends the administration of a full course of SP to infants below 12 months during routine DTP and vaccination against measles administered through Expanded Program on Immunization (EPI) in areas with moderate-to-high malaria transmission (annual entomological inoculation rate of greater than 10) [1]. Given the high level of resistance to SP in most African settings, this policy should only be applied in areas where the prevalence of* Pfdhps* 540 mutations is less than or equal to 50% as the* Pfdhps* K540E mutation is a surrogate marker for the quintuple mutation conferring resistance to SP [1]. Our results showed that the* Pfdhps* 540 mutation was seen in just 5 patients in 2003–2005 and in 2009–2013 and the mutation was absent. The policy of sulphadoxine-pyrimethamine-intermittent preventive therapy in infants (SP-IPTi) and pregnant women (SP-IPTp) should be reenforced in the area given its potential benefits.

This study was limited to Mutengene in the South West Region and further analysis of the present situation using data from other regions of the country can give a better picture of the evolutionary dynamics of drug-resistant mutants in the country in the face of changing therapy. Moreover, only key SNPs were considered in the study and analysis of the other SNPs in these marker genes will throw more light on the evolutionary dynamics.

## 6. Conclusions

This study showed that the evolution of treatment policies in Cameroon had led to the gradual return of the sensitive genotype of the 4-aminoquinoline resistance markers. The antifolate resistance markers genotype on the other hand (except the* Pfdhps* K540E) increased to saturation. Moreover, the gradual return to the CQ-sensitive genotype showed that return to its clinical efficacy can be anticipated as was the case in Malawi.

## Figures and Tables

**Table 1:** Changes in the frequency of *Pfcrt*, *Pfmdr1*, *Pfdhfr*, and *Pfdhps* genotypes between the study periods.

Gene	Mutation	Notation	2003–2005	2009–2013	95% CI	*P* value
*Pfcrt*	K76T	T	96.1 (223/230)	66.9 (174/260)	22.96–35.44	***P < 0.0001***
		K	3.9 (7/230)	33.1 (84/260)		

*Pfmdr1 *	N86Y	Y	83.6 (189/226)	45.2 (94/208)	30.09–46.71	***P < 0.0001***
		N	16.4 (37/226)	54.8 (116/208)		
	Y184F	F	97.3 (217/223)	56 (131/234)	30.09–46.71	***P < 0.0001***
		Y	2.7 (6/233)	44 (103/234)		
	D1246Y	Y	3.1 (7/226)	0 (0/250)	0.84–5.36	***0.02***
		D	96.9 (219/226)	100 (250/250)		

*Pfdhfr*	N51I	N	3.6 (08/222)	1.2 (3/243)	−0.41–5.21	*0.16*
		I	96.4 (214/222)	98.8 (240/243)		
	C59R	C	2.3 (05/222)	0 (0/243)	0.33–4.27	*0.05*
		R	97.7 (217/222)	100 (243/243)		
	S108N	S	0.9 (2/222)	0 (0/243)	−0.34–2.14	*0.44*
		N	99.1 (220/222)	100 (243/243)		

*Pfdhps*	A437G	A	13.3 (30/225)	10 (24/240)	−2.54–9.14	*0.33*
		G	86.7 (195/225)	90 (216/240)		
	K540E	K	97.8 (220/225)	100 (240/240)	0.28–4.12	*P = 0.06*
		E	2.2 (5/225)	0 (0/240)		

Letters represent single letter representation of amino acids in the codons of the drug resistance genes. CI = confidence interval. In the above table, mixed infections are considered to have the mutant genotype.
